# Premature termination codons in *SOD1* causing Amyotrophic Lateral Sclerosis are predicted to escape the nonsense-mediated mRNA decay

**DOI:** 10.1038/s41598-020-77716-5

**Published:** 2020-11-26

**Authors:** Claire Guissart, Kevin Mouzat, Jovana Kantar, Baptiste Louveau, Paul Vilquin, Anne Polge, Cédric Raoul, Serge Lumbroso

**Affiliations:** 1 Laboratoire de Biochimie et Biologie Moléculaire, CHU Nimes, University of Montpellier, Nimes, France; 2 The Neuroscience Institute of Montpellier, INM, INSERM, Univ Montpellier, Montpellier, France; 3 Département de Pharmacologie et de Génomique des Tumeurs Solides, Hôpital Saint Louis, Assistance Publique Hôpitaux de Paris, Paris, France

**Keywords:** Genetics, Medical genetics, Motor neuron disease, Amyotrophic lateral sclerosis, Molecular medicine, RNA decay

## Abstract

Amyotrophic lateral sclerosis (ALS) is the most common and severe adult-onset motoneuron disease and has currently no effective therapy. Approximately 20% of familial ALS cases are caused by dominantly-inherited mutations in the gene encoding Cu/Zn superoxide dismutase (*SOD1*), which represents one of the most frequent genetic cause of ALS. Despite the overwhelming majority of ALS-causing missense mutations in *SOD1*, a minority of premature termination codons (PTCs) have been identified. mRNA harboring PTCs are known to be rapidly degraded by nonsense-mediated mRNA decay (NMD), which limits the production of truncated proteins. The rules of NMD surveillance varying with PTC location in mRNA, we analyzed the localization of PTCs in *SOD1* mRNA to evaluate whether or not those PTCs can be triggered to degradation by the NMD pathway. Our study shows that all pathogenic PTCs described in *SOD1* so far can theoretically escape the NMD, resulting in the production of truncated protein. This finding supports the hypothesis that haploinsufficiency is not an underlying mechanism of *SOD1* mutant-associated ALS and suggests that PTCs found in the regions that trigger NMD are not pathogenic. Such a consideration is particularly important since the availability of *SOD1* antisense strategies, in view of variant treatment assignment.

## Introduction

Amyotrophic lateral sclerosis (ALS) is a fatal neurodegenerative disease characterized by the selective loss of both upper and lower motoneurons, leading to a progressive paralysis and death within 3–5 years^1^.

About 20% of familial ALS cases are caused by mutations in the gene encoding the detoxifying copper-zinc superoxide dismutase (*SOD1*)^2^. Currently, over 180 different mutations throughout the five exons of the *SOD1* gene (MIM 147450) have been described^2,3^, the vast majority of which being missense point mutations resulting in a dominant mode of inheritance of ALS (with the exception of the D91A mutation) and spreading over the entire 154 amino acid sequence^4,5^.

It has been well-established that *SOD1* mutants-mediated toxicity is caused by a gain-of-function rather than the loss of the detoxifying activity of SOD1^2^ and that mutant SOD1 can adopt multiple misfolded conformations that mediate toxicity^2^. Moreover, mice with genetic ablation of *Sod1* do not recapitulate disease phenotype^5–8^. Instead, *Sod1*-deficient mice show accelerated rate of muscle denervation, locomotor deficits and tremors, as well as increased vulnerability to stress. It is noteworthy that the 50% loss of Sod1 activity described in heterozygous *Sod1*^+*/-*^ mice leads to an increased susceptibility to axonal injury, ischemia or glutamate-induced toxicity^9^.

Nonsense-mediated mRNA decay (NMD) is an eukaryotic quality control pathway that degrades mRNAs containing Premature termination codons (PTCs) caused by nonsense or frameshift mutations^10,11^. It is important to note that some PTCs can escape NMD. This capability is governed by four rules^12^: (1) the 50 nucleotides rule: PTCs less than 50–55 nucleotides upstream of the last exon–exon junction typically do not trigger NMD; (2) the last exon rule: PTCs in the last exon of a gene also do not trigger NMD; (3) the long exon rule: exons greater than approximately 400 nucleotides inhibit NMD; (4) the start-proximal rule: PTCs located below 150 nucleotides from the start codon typically fail to trigger NMD.

PTCs that escape NMD in *SOD1* are thus expected to lead to the production of truncated SOD1 protein, which can be highly unstructured with elevated toxicity as illustrated with the non-sense mutation p.Leu127*^13,14^.

To evaluate the capacity of PTCs associated with ALS in *SOD1* to trigger NMD, we analyzed their localization through the gene.

## Results

**Figure 1:**
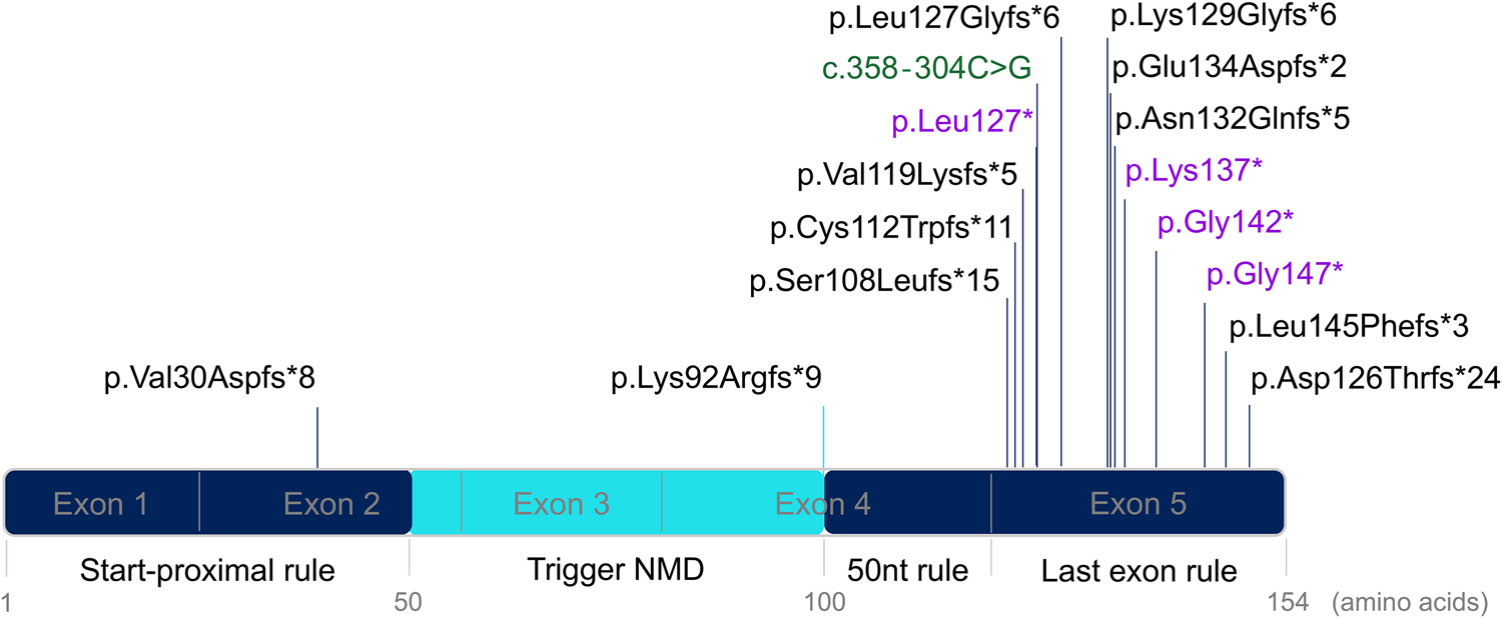
Schematic representation of the *SOD1* gene showing the localization of PTCs causing ALS and NMD escaping regions*.* Frameshift mutations and the deep intronic splicing mutation are placed according to the localization of their resulting PTCs (and not the mutation site itself). Nonsense mutations are indicated in purple, deep intronic splicing mutation in green and frameshifts mutations in black. The region of *SOD1* where PTCs trigger NMD is represented in cyan. The regions that escape NMD are represented in dark blue.

Regarding the NMD rules we estimated that the region of *SOD1* obeying the NMD is located between nucleotides 151–301, which correspond to amino acids 50–100 (Fig. 1). Accordingly, all PTCs located within this region are expected to trigger the NMD, resulting in the degradation of messenger RNAs, and leading to haploinsufficiency. Conversely, all PTCs located outside this region can result in the production of truncated SOD1, prone to induce misfolded protein. We found a total of 16 disease-associated-PTCs mutations in *SOD1* in the literature^15,16^, including 4 nonsense mutations, 11 frameshift mutations and 1 deep intronic splicing mutation (Table 1, Fig. 1).

**Table 1:** List of truncating mutations in *SOD1* associated with ALS.

Mutation (GRCh37)^a^	Protein variant ^b^	Resulting PTC position	References
Splicing mutation			
c.358-304C > G	p.Val120Glnfs*8	127	^19^
Non-sense mutations			
c.380 T > A	p.Leu127*	127	^16^
c.409A > T	p.Lys137*	137	^15^
c.424G > T	p.Gly142*	142	^36^
c.441 T > A	p.Cys147*	147	^20,37^
Frameshift mutations			
c.88_89insA	p.Val30Aspfs*8	37	^20,38,39^
c.275_276delAA	p.Lys92Argfs*9	100	^40^
c.320dupT	p.Ser108Leufs*15	122	^41^
c.335dupG	p.Cys112Trpfs*11	122	^28,29^
c.355delGinsAAAAC	p.Val119Lysfs*5	123	^20,42^
c.379_380delTT	p.Leu127Glyfs*6	132	^43–45^
c.380_383dupTGGG	p.Lys129Glyfs*6	134	^20,46,47^
c.401_402insTT	p.Glu134Aspfs*2	135	^20,48^
c.383_392dupGCAAAGGTGG	p.Asn132Glnfs*5	136	^49^
c.435delGinsCGTTTA	p.Leu145Phefs*3	147	^50^
c.376delG	p.Asp126Thrfs*24	149	^51^

^a^ Human genome variation society (HGVS) nomenclature V2.0 according to mRNA reference sequence GenBank: NM_000454.4. Nucleotide numbering uses + 1 as the A of the ATG translation initiation codon in the reference sequence, with the initiation codon as codon 1. ^b^ HGVS nomenclature according to protein reference sequence GenPept: NP_000445.1. Amino acid numbering uses p.1 as the Methionine corresponding to the initiation codon. This implies a 1-amino acid switch compared to former *SOD1* nomenclature (*eg.* L127X mutation was formerly known as L126X or, in some articles, L126Z).

Among them, fourteen are predicted to escape the NMD according to the last exon rule and one (p.Val30Aspfs*8) is predicted to escape the NMD, obeying to the start proximal rule (Fig. 1). By faithfully following the 50 nucleotides rule, one frameshift mutation (p.Lys92Argfs*9) was found to introduce a PTC at the position 100, which is the last position predicted to trigger NMD. However, it is likely that at such borderline position, NMD is not completely activated and that truncated protein is at least partly produced.

**Figure 2:**
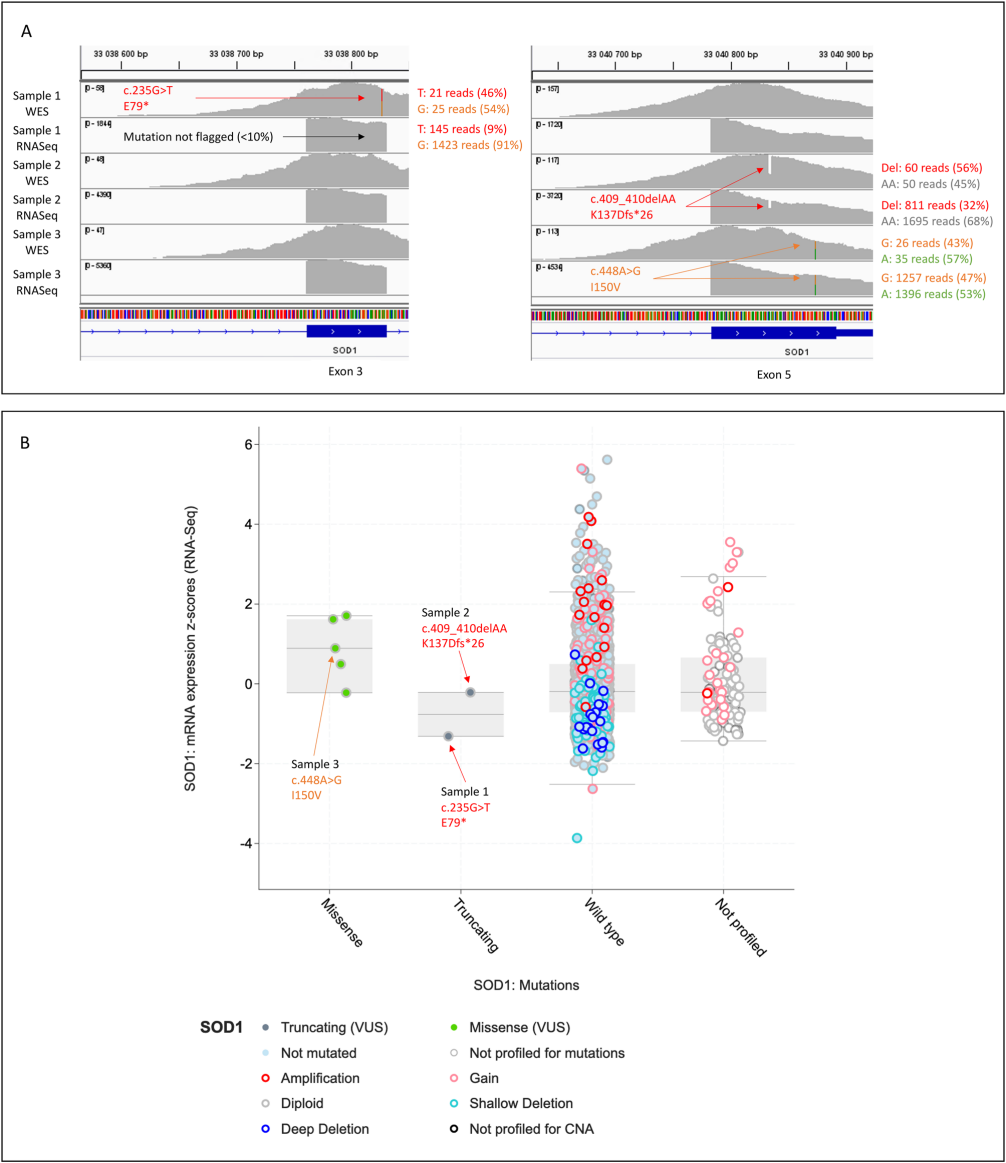
(**A**) Evaluation of NMD effect on *SOD1* transcripts by comparison of matched tumor exomes and transcriptomes. Visualization with the Integrative Genome Viewer software (version 2.8.4, http://software.broadinstitute.org/software/igv/)^35^ of WES and RNA-Seq alignments of 3 samples from the Cancer Cell Line Encyclopedia^52^. The missense mutation p.Ile150Val (I150V, sample 3) is used here as a control as it has no effect on NMD activity and is thus found heterozygous in both WES and RNA-Seq alignments. The non-sense mutation p.Glu79* located in exon 3 of *SOD1*, (E79*, sample 1) was detected on 9% of RNA-Seq reads versus 46% of WES reads, showing the degradation of the *SOD1* mutated transcript by NMD. In contrast, heterozygous frameshift mutation p.Lys137Aspfs*26 (K137Dfs*26, sample 2), located in the last exon of *SOD1* was detected in 32% of RNA-Seq reads versus 56% of WES reads, highlighting NMD escape. (**B**) *SOD1* mRNA expression correlation with *SOD1* mutations in 2029 samples. This plot was generated from the cBio Cancer Genomics Portal (http://cbioportal.org)^17,18^. Although not statistically significant, sample 1 with the E79* mutation appears to have a lower *SOD1* mRNA expression (z-score = −1.31) compared to sample 2 with the K137Dfs*26 mutation (z-score = −0.21, equivalent to the mean mRNA expression in the wild type group). Deep Deletion indicates a deep loss, possibly a homozygous deletion; Shallow Deletion indicates a shallow loss, possibly a heterozygous deletion; Gain indicates a low-level gain (a few additional copies, often broad); Amplification indicate a high-level amplification (more copies, often focal); Not profiled for CNA indicate the samples for which copy-number analysis was not performed. These levels are derived from copy-number analysis algorithms and indicate the copy-number level per gene.

To evaluate the impact of NMD on PTCs across the *SOD1* gene, we have analyzed experimental data from a large-scale analysis of approximately 80,000 matched tumor exomes and transcriptomes available on the cBio Cancer Genomics Portal^17,18^. Among them, we identified 31 mutations in *SOD1* in 40 samples. 24 were missense mutations, 2 were translation start site mutations and 5 were truncating mutations (2 splicing, 2 frameshift and 1 non-sense mutation). The heterozygous non-sense mutation (p.Glu79*, E79*) identified in one sample by whole exome sequencing (WES) was almost not detected on the RNA sequencing (RNA-Seq) data from the same sample, suggesting a massive degradation of the *SOD1* mutated transcript by the NMD (Fig. 2). In contrast, heterozygous frameshift mutation (p.Lys137Aspfs*26, K137Dfs*26), located in the last exon of *SOD1*, was detected in both WES and RNA-Seq data, confirming NMD escape for this other PTC (Fig. 2).

## Discussion

In this study, we have explored the impact of NMD on *SOD1* and shown that the activity of the NMD pathway is of broad importance for ALS caused by PTC in *SOD1*. Through a large-scale analysis of human cancer exomes and transcriptomes we were able to confirm that *SOD1* standardly obeys to the NMD pathway and its rules.

Our conclusion is supported by several arguments from the literature: (1) The presence of PTC in a region that escape NMD has been detected in mRNA extracted from immortalized lymphoblast cell lines from two patients harboring the c.358-304C > G mutation, thus confirming the impact on the protein level p.Val120Glnfs*8^19^; (2) Conformational changes of truncated proteins have been well characterized for PTC located in the region that escape NMD^20^; (3) Heterozygous PTCs located in the region that trigger NMD seem to be more frequent in individuals from general population: in gnomAD database, for example, we found 6 individuals with a PTC theoretically triggering NMD (p.Glu50Glyfs*39, p.Leu68Glufs*19) versus 3 individuals aged between 40 and 65 years with a PTC theoretically escaping NMD (p.Val6Cysfs*4, p.Asp97Metfs*8).

Interestingly, the fact that all PTCs associated with ALS in *SOD1* can escape the NMD comforts the hypothesis that haploinsufficiency is not an underlying mechanism of the disease. Instead, the production of a misfolded truncated SOD1 protein could cause a toxic gain-of-function. Therefore, even if we confirmed the massive degradation of mRNA harboring a PTC in the region triggering NMD, as we could detect a small remaining amount of mutated mRNA (9% of reads on RNA-Seq data, see sample 1 on Fig. 2A), we cannot exclude a very late onset form of SLA in such situation.

Dimer destabilization, oligomerization and increased aggregation are the proposed mechanisms for mutant SOD1 toxicity^4^. Recently it has been demonstrated that SOD1 acts as a H_2_O_2_-responsive regulatory protein in the expression of ALS-linked genes. Both sequence preference and affinity of SOD1 interactions with DNA depend on SOD1 conformation^21^. Thereby, PTCs that escape NMD in *SOD1* are expected to cause toxic conformational changes. Indeed, some of the truncating mutations described here were proven to cause SOD1 misfolding capable to interact with Derlin-1, triggering endoplasmic reticulum stress and contributing to motoneuron death (*i.e.* p.Val30Aspfs*8, p.Val119Lysfs*5, p.Leu127*, p.Glu134Aspfs*2, p.Gly142*)^20^.

The toxic gain-of-function mechanism evidence provides a strong rationale for gene silencing as a therapy for *SOD1*-mediated ALS. Thus, clinically promising therapies, all aimed at enhancing specifically the degradation of the mutated *SOD1* RNA, such as anti-sense oligonucleotides (ASO) and RNA interference (RNAi) are being tested in preclinical and clinical studies^22–25^. In the first clinical trial of ASO treatment in human beings, only ASO targeting missense mutations were developed. This trial had favorable safety outcomes, and a trial to assess the safety, tolerability and pharmacokinetics of a second generation *SOD1* ASO is currently in progress (ClinicalTrials.gov, NTC02623699)^26^. To our knowledge, no ASO targeting a PTC has been investigated so far. This could be explained by the low proportion of patients carrying such mutations. For example, PTCs in *SOD1* are absent from MinE Database which includes 4366 whole genomes from ALS patients and 1832 whole genomes from controls, from different European ancestry^27^.

Recently, complete loss of function of *SOD1* in human has been reported in a 2 years old girl with a homozygous truncating mutation and an absence of SOD1 activity. The patient presented with axial hypotonia and loss of gross and fine motor function at 6 months of age, after which severe, progressive spastic tetraparesis developed and Babinski’s sign was present in both feet. Atrophy, fasciculations, and other signs of lower motor neuron involvement were not noted. Her parents, both heterozygous for the mutation were healthy at the time of the report while the level of SOD1 activity was half that of the normal level^28^. Another report of the same homozygous truncating variant c.335dupG (p.Cys112Trpfs*11) in *SOD1* was identified in another patient with tetraspasticity. In contrast with Andersen et al. 2019 report, heterozygous carriers from this family had a markedly reduced enzyme activity when compared to wild-type controls but show no overt neurologic phenotype^29^. Thus, while caution might be exercised regarding the use of gene therapies that may markedly depresses SOD1 activity, reduction of SOD1 appears to be well tolerated, as outlined by the favorable clinical trial safety outcomes.

Animal models, particularly SOD1 rodent model^30^, initially developed to investigate the complex processes occurring in ALS, had played a major role in performance evaluation of these silencing approaches^22^. More recently, other models like zebrafish^31^, Drosophila^32^ or patient-derived induced pluripotent stem cell^33^ have also been designed and tested to investigate the physiopathology of ALS. These models, particularly useful since the availability of *SOD1* antisense strategies, offer the possibility to study the pathogenicity of novel *SOD1* variants, especially complex intronic mutations that could either lead to an amino-acid(s) insertion or deletion and/or to the creation of a PTC.

In conclusion, we highlight that all described PTCs in *SOD1* causing ALS are predicted to escape the nonsense-mediated mRNA decay. More importantly, this observation suggests that truncating mutations found in the region of *SOD1* that trigger NMD may have no pathogenic significance. Such a consideration is particularly important since the availability of *SOD1* antisense strategies, in view of variant treatment assignment.

## Methods

The PTCs of the human *SOD1* gene (NM_000454.4) resulting from nonsense, frameshift and splicing mutations that are associated with ALS were obtained from the Human gene mutation database (HGMD)^34^, which provides systematic and in-depth qualitative and quantitative overviews of genetic research in both familial and sporadic ALS. Intronic mutations located outside the canonical sites and not confirmed by transcript analysis were excluded from this study.

The cBio Cancer Genomics Portal (http://cbioportal.org)^17,18^, an open platform for exploring multidimensional cancer genomics data, was used to select tumor samples with PTC in *SOD1* for which whole exome sequencing (WES) and RNA-sequencing (RNA-seq) experimental data were performed. We generated plot from cBio Cancer Genomics Portal^17,18^ to analyze *SOD1* mRNA expression correlation with *SOD1* mutations.

Raw data (Bam files) from sample of interest were downloaded, when available, from NCBI Sequence Read Archive (SRA) and visualized with the Integrative Genome Viewer software (version 2.8.4, http://software.broadinstitute.org/software/igv/)^35^. SRA accession numbers and detailed information about the samples are available in the Supplementary Table S1.

## Supplementary information

Supplementary Information.
